# Sciatica

**Published:** 1891-02-14

**Authors:** 


### The Practitioner's Mirror and Retrospect.

[*The Editor will be glad to receive offers of co-operation and contributions from members of the profession. All letters should be addressed to* The Editor, The Lodge, Porchester Square, London, W.]

### MEDICINE.

### SCIATICA.

It is certain that under the term sciatica are included at least two distinct pathological conditions. The term sciatica has been made to include all neuralgias in the district of the sciatic nerve indiscriminately. Recently, however, a more accurate pathological study of the affection has tended not only to differentiate between true sciatica and conditions which have been erroneously regarded as sciatica, but also to distinguish pathological varieties of sciatica properly so-called. It is hardly necessary to insist on the importance of such investigations as bearing on success in the treatment, and we cannot be surprised that, however successful certain methods proved in the one class, when applied indiscriminately, they one and all are disappointing. The rheumatic and gouty diatheses undoubtedly predispose to sciatica, and muscular rheumatism and gouty affections are frequently diagnosed as disease of the sciatic nerve. Dr. Buzzard states that there are three conditions of the muscular system which are apt to be confounded with sciatica, viz.—(1) Myalgia, from over-exertion of the flexor muscles at the back of the thigh. The pain is most marked at the points of insertion of the muscles, and is only felt when the muscles act. (2) Muscular rheumatism, the result of exposure to cold. In these cases, too, the pain is only felt during muscular action, a point that is well observed in lumbago. In sciatica the pain is more or less continuous, and is independent of muscular action. (2) A low inflammation of the loose bursal tissue which separates the large muscles of the thigh, brought about by the presence of some morbid material conveyed by the lymphatic vessels with which the spaces are in direct communication. Uric acid and pus, he writes, are especially liable to cause this affection. Other diseases which are liable to be mistaken for sciatic neuralgia are sacroiliac disease, hip-joint disease, the lightning pains of locomotor ataxia, and the darting pains of renal calculus.

Dr. Pritchard, in a recent article (*Amer. Jour. of Med. Sci.*, Jan.), indicates the points of distinction between the varieties of true sciatica. There are two well-marked pathological varieties: (1) neuritis of the sciatic nerve, in which definite pathological changes, chiefly infiltration with leucocytes, and other characteristic inflammatory lesions, are observable in the nerve structures after death, and (2) sciatic neuralgia, in which no obvious pathological change has been observed, and which, in the present state of pathology, can only be attributable to abnormal trophic states of the nerve, and may be best expressed by the term functional. Clinically, the causes of neuritis are found to be chiefly wounds, and tumours pressing on the nerve trunk; of sciatic neuralgia the causes are the same, excepting that constitutional states, as anæmia, malaria (?), indicate neuralgia, unless bilateral. The pain of neuritis is duller and more paroxysmal than in neuralgia, and paræsthesiæ of pricking and numbness, especially numbness, are rarer in neuralgia. Movement of the limb, especially forcible extension, is generally found to increase the pain in neuritis, while the pain of neuralgia is not specially aggravated, if at all affected, by movement. Anæsthesia with, at times, rapid onset and limited in area, in neuritis, while actual anæsthesia is very rare in neuralgia, though general numbness may occur. In neuritis a certain amount of swelling along the course of the nerves, and tenderness on pressure; in neuralgia no swelling nor tenderness, but pain on pressure at certain points. In neuritis wasting of the muscles and trophic changes in the skin, hair, and nails; in neuralgia no trophic changes, but slight wasting only from disuse, and whereas in the former paresis or paralysis of muscles, with reaction of degeneration to galvanic current, may be present, in the latter these are not observed. Such, then, are the chief distinguishing characteristics of sciatic neuritis and sciatic neuralgia, and the great importance of their differential diagnosis becomes evident when we remember that they are practically distinct diseases, each calling for special forms of treatment. The treatment should be constitutional as well as local. Of course, if the disease is of rheumatic, gouty, or syphilitic origin, appropriate remedies at once suggest themselves; if of malarial origin, quinine is called for. It is often necessary to resort to injections of morphine or atropine, either alone or in combination.

In neuritis iodide of potassium and Rhus toxicodendron are useful drugs. Dr. Pritchard has found massage give favourable results. Massage was recommended by Dr. Schüller in 1886, and he gives the following directions for its application in this affection: "The patient lies on his good side, the hip and knee-joints being slightly bent. The rubbing is done upwards along the course of the sciatic nerve, now with both thumbs, now with the ball of the little finger or thumb; then the part is knocked with the closed fist, and then the muscles are pressed down over the nerve with both hands, kneaded, etc." It need hardly be remarked that such procedure gives rise to considerable pain, but this soon passes off, the neuralgic pains become less marked, and after each operation the patient finds walking easier. Dr. Schüller found that massage was most particularly successful in sciatica resulting from chills and injuries. In sciatic neuralgia the results were less encouraging. The time required for recovery varied between nine days and five and a-half weeks. Where the disease has had a rheumatic origin we have found salol, salicylic acid, salicylate of soda, and salicine give relief, and in many cases cure. The dithiosalicylate of soda is disappointing in sciatic neuritis. If the reaction of degeneration be present, it is necessary to employ the galvanic current to prevent the wasting of the muscles as far as possible; this form of electricity will often relieve the pain. In sciatic neuralgia arsenic should be administered, and may be advantageously combined with iron and cod liver oil. Phenacetine, antipyrin, antifebrin, and exalgine have all been used with some success. In cases where the stomach becomes intolerant of salicylate of soda Weiss has recommended smaller doses combined with ten grains of phenacetin or antipyrin. It is difficult to indicate the cases in which exalgine will succeed. As a rule it is disappointing, but in few it proves an excellent analgesic.

We have already referred to hypodermic injections of morphine for the relief of pain, but these injections are sometimes followed by a definite and continued improvement in the disease. How it acts therapeutically is not known. Dr. S. Solis-Cohen reports a case in which, nerve stretching and many other varieties of treatment having been adopted without avail, a one-per-cent. solution of osmic acid injected into the tissues near the the exit of sciatic nerve proved successful. He began by using ten minims, increasing the dose of the injections until he finally gave thirty minims at a single injection. At first the injections were made daily, and later three times a-week, the treatment lasting seven weeks. Osmic acid, thus injected, gives considerable pain, and abscesses are rather liable to form at the seat of injections. It should be used only as a last resort. Banduy and Mays have used hypodermic injections of theine. The average dose is half a grain, but Mays has injected as much as three grains. Theine seems to possess some analgesic properties. Counter-irritation is an old method of treatment. This can be induced by the use of the actual cautery along the course of the nerve, or by the application of blisters. Chloride of methyl applied in the form of a spray has been recommended by Dr. Debove. The spray is applied to the whole area over which the pain is felt; the skin instantly freezes, growing white and hard like a stone, and the patient experiences a sensation similar to that produced by the application of the actual cautery. An erythema usually appears on the frozen parts. One of the best forms of counter-irritation is the faradic current applied with the electric brush along the course of the nerve. Nerve stretching was at one time warmly advocated by a few, but it has almost dropped out of use. Nerve-stretching by flexion of the thigh, and by suspension, are simple methods that may give relief at times. Turkish baths, and the waters of Bath, Buxton, and Wildbad, prove beneficial in some of the more chronic cases.

